# Diagnostic accuracy of growth hormone stimulation tests for growth hormone deficiency following radiotherapy involving the hypothalamic-pituitary axis: protocol for a systematic review and planned meta-analysis

**DOI:** 10.1136/bmjopen-2026-125247

**Published:** 2026-09-21

**Authors:** Lillian Ruiheng Chen, Melissa Betty Perry, Maria Salomon Estebanez, Daniel Livesey, Glen Philip Martin, Claire E Higham

**Affiliations:** 1 Department of Endocrinology, The Christie NHS Foundation Trust, Manchester, UK; 2 Christie Patient Centred Research, The Christie NHS Foundation Trust, Manchester, UK; 3 Department of Paediatric Endocrinology, Royal Manchester Children’s Hospital, Manchester, UK; 4 The Christie Institute for Cancer Education, The Christie NHS Foundation Trust, Manchester, UK; 5 Division of Informatics, Imaging and Data Science, The University of Manchester, Manchester, UK; 6 Department of Endocrinology, The Christie Hospital NHS Trust, Manchester, UK; 7 Division of Cancer Sciences, The University of Manchester, Manchester, UK; 8 Manchester Biomedical Research Centre (BRC), NIHR, Manchester, UK

**Keywords:** Cancer Survivors, RADIOTHERAPY, Diabetes & endocrinology, Pituitary disorders, Systematic Review, Sensitivity and Specificity

## Abstract

**Abstract:**

**Introduction:**

Radiotherapy is part of the treatment for many intracranial tumours, head and neck cancers and haematological malignancies. In these cases, the hypothalamic-pituitary (HP) axis is often included within the radiation field and can be damaged as an organ at risk. As survival improves, an increasing number of patients are living with late endocrine complications, particularly growth hormone deficiency (GHD). Diagnosing GHD in this context is challenging, as much of the evidence supporting growth hormone (GH) stimulation tests comes from populations with fundamentally different disease mechanisms. We aim to conduct a systematic review, and meta-analysis where feasible, to assess the diagnostic accuracy of GH stimulation tests for diagnosing GHD in children, teenagers, young adults and adults who have received radiotherapy involving the HP axis.

**Methods and analysis:**

Eligible studies will include patients exposed to radiotherapy involving the HP axis, who subsequently underwent GH stimulation testing for suspected GHD. Index tests will include commonly used stimulation tests such as the insulin tolerance test, glucagon stimulation test, arginine stimulation test, clonidine stimulation test, GH-releasing hormone-arginine stimulation test and macimorelin stimulation test. Reference standards will include clinical criteria (≥2 additional pituitary hormone deficiencies) or alternative GH stimulation tests. Searches will be conducted in MEDLINE, Embase, Cochrane Library and PubMed. Two independent reviewers will screen studies, collect data, evaluate risk of bias using Quality Assessment of Diagnostic Accuracy Studies-2 and assess reporting quality using the Standards for Reporting of Diagnostic Accuracy Studies criteria. Sensitivity and specificity will be extracted. Where studies are sufficiently comparable, results will be pooled using hierarchical bivariate and summary receiver operating characteristic models. Prespecified subgroup analyses will explore heterogeneity by age group, body mass index, ethnicity, radiotherapy dose and time since treatment.

**Ethics and dissemination:**

Ethical approval is not required as this systematic review summarises published data only. We will share our findings through peer-reviewed publications, conference presentations and engagement with relevant clinical teams to improve diagnostic decision-making in our population of interest.

**PROSPERO registration number:**

CRD420261415689.

**STRENGTHS AND LIMITATIONS OF THIS STUDY**

- This will be the first systematic review and meta-analysis where feasible, specifically evaluating the diagnostic accuracy of growth hormone stimulation tests in patients exposed to radiotherapy involving the hypothalamic-pituitary axis.
- A robust and peer-reviewed search strategy will be applied across four major databases.
- Inclusion of studies published only in English, French and Spanish may introduce language bias.
- Reporting quality of included diagnostic accuracy studies will be assessed using the Standards for Reporting of Diagnostic Accuracy Studies criteria, alongside methodological quality assessment using Quality Assessment of Diagnostic Accuracy Studies-2.
- Heterogeneity across studies in terms of patient populations, stimulation test protocols, diagnostic thresholds and reference standards may limit the feasibility of meta-analysis.

## Introduction

 Radiotherapy is an integral component of treatment for many intracranial tumours, head and neck cancers and haematological malignancies.^1–3^ In these cases, the hypothalamic-pituitary (HP) axis is often included within the radiation field and can be damaged as an organ at risk. As long-term survival continues to improve, an increasing number of these patients are living with the late endocrine consequences of radiotherapy involving the HP axis.^4 5^ Growth hormone (GH) deficiency (GHD) is among the most common late effect in this population, which reflects the vulnerability of the somatotropic axis to radiation-induced damage.^4 6^ Among childhood cancer survivors exposed to radiotherapy involving the HP axis, GHD has been reported in up to 50–100% of patients at 5–15 years after radiotherapy.^7–10^ In those treated as adults, approximately 30% developed GHD over the years.^11 12^ GHD remains substantially underdiagnosed and undertreated among this population, which likely contributes to the wide variation in reported prevalence across studies.^7–12^ In one large US report, 99.7% of survivors with GHD following cranial radiotherapy were not receiving GH replacement therapy.^13^

In children, GHD typically presents with short stature and reduced growth velocity.^14^ In adults, GHD is associated with central obesity, increased fat mass, reduced lean muscle mass, insulin resistance, higher fracture risk and decreased quality of life.^15^

The standard treatment for both paediatric and adult GHD is recombinant human GH, usually given as daily injections. Longer-acting weekly formulations are available, but their higher cost has limited their use. Additionally, the safety of the long-acting formulations in cancer or tumour survivors is unknown. In children, recombinant human GH leads to substantial gains in final adult height. In teenagers and adults, treatment improves body composition, bone health and quality of life.^16^ Importantly, current evidence does not support an association between daily GH replacement therapy and increased cancer-related mortality, primary tumour development or cancer recurrence in GHD survivors.^17^ On this basis, consensus guidelines^17^ recommend considering GH replacement in childhood cancer or intracranial tumour survivors when growth velocity declines and GHD is confirmed biochemically, with the timing of treatment individualised based on clinical status, tumour type and treatment history. In adults, GH replacement may also be offered to head and neck cancer or intracranial tumour survivors with confirmed GHD who are in remission, after careful case-by-case assessment.^17^

Accurate diagnostic tests are essential to avoid misdiagnosis. An accurate diagnosis is crucial to avoid unnecessary, burdensome and costly long-term treatment and its potential side effects, while ensuring that those with true GHD are not denied a therapy from which they may benefit.

Diagnosing GHD is inherently challenging. Because GH secretion is pulsatile, random serum measurements are unreliable. The level of insulin-like growth factor 1 (IGF-1) reflects GH activity, but cannot be used as a standalone marker, particularly in radiotherapy-induced GHD^18–20^ where a low IGF-1 level strongly suggests GHD, but normal IGF-1 concentrations are frequently observed in individuals with true GHD.^21 22^ Therefore, in most cases, diagnosis instead relies on a composite of clinical assessment and GH stimulation test results^16 18 19^ (table 1). GH stimulation tests are limited by significant methodological heterogeneity across existing literature, including the use of arbitrary diagnostic cut-offs, varying test durations, inconsistent dosing of stimulating agents and different sampling time points. Furthermore, the assessment of diagnostic accuracy of these tests is confounded by high intra-assay and inter-assay variability, and the absence of a ‘true’ gold standard. Finally, many studies assessing GH stimulation tests were published prior to the introduction of the Standards for Reporting of Diagnostic Accuracy Studies (STARD) Guidelines, which were developed to standardise and improve the reporting of diagnostic accuracy studies.^23^ Consequently, the quality and completeness of reporting across the literature may be variable.

**Table 1:** Characteristics of commonly used growth hormone stimulation tests for the diagnosis of growth hormone deficiency

Characteristics	Insulin tolerance test	Glucagon stimulation test	Arginine stimulation test	Clonidine stimulation test	GHRH+arginine	Macimorelin stimulation test
Approved population	Children and adults	Children and adults	Children	Children	Adults	Adults
Mechanism of action	Induced hypoglycaemia stimulates hypothalamic release of GHRH	Not fully understood	Suppresses hypothalamic secretion of somatostatin, leading to increased pituitary release of GH	Stimulates hypothalamic release of GHRH	GHRH directly stimulates the pituitary release of GH and arginine suppresses hypothalamic secretion of somatostatin	Stimulates hypothalamic and pituitary release of GH
Assessment of	Whole hypothalamic-pituitary-somatotroph axis	Whole hypothalamic-pituitary-somatotroph axis	Pituitary alone	Whole hypothalamic-pituitary-somatotroph axis	Pituitary alone	Uncertain (whole axis or pituitary alone)
Described adverse effects	Symptomatic hypoglycaemia Seizures Concerns over risk of cardiovascular events during severe hypoglycaemia	Nausea Vomiting Headache Severe hypotension	Rare cases of transient haematuria	Hypoglycaemia Hypotension Fatigue	Transient facial flushing	Mild dysgeusia Concerns over risk of QTc prolongation particularly with concomitant use of QT-prolonging medications
Advantages	Historically accepted as the gold standard Allows concurrent assessment of the hypothalamic-pituitary-adrenal axis	Generally considered safe and reproducible Allows concurrent assessment of the hypothalamic-pituitary-adrenal axis	Generally considered safe	Generally considered safe Oral administration Good specificity	Generally considered safe	Generally considered safe Oral administration Short test duration
Disadvantages	Frequent adverse effects Safety concerns Limited reproducibility Requires close medical supervision Contraindicated in elderly patients and those with cardiovascular disease, stroke or seizure disorders	Frequent adverse effects Long test duration	Limited reproducibility Higher false-positive rate	Limited reproducibility	Currently unavailable in Europe and North America	Currently unavailable in North America
Availability	Yes	Yes	Yes	Yes	GHRH is currently unavailable in Europe and North America	Yes in Europe; currently unavailable in North America

GH, growth hormone; GHRH, GH-releasing hormone.

In adults, much of the evidence supporting the GH stimulation tests comes from patients with pituitary or suprasellar tumours, which are often benign.^24–26^ These studies rarely report whether participants had previous radiotherapy involving the HP axis, and when they do, they seldom examine how test results differ between irradiated and non-irradiated patients.^27 28^ The underlying pathophysiology of GHD differs substantially between these populations. Pituitary or suprasellar tumours and their surgical treatment typically cause direct pituitary damage, whereas radiotherapy is thought to primarily affect the hypothalamus,^29–31^ although evidence is limited. This raises important concerns regarding the diagnostic accuracy of existing GH stimulation tests in patients exposed to radiotherapy involving the HP axis. For example, tests such as the GH-releasing hormone (GHRH)-arginine test predominantly stimulate the pituitary and may yield false-negative results in individuals with hypothalamic damage but preserved pituitary function. In fact, the Endocrine Society’s 2011 guidelines on adult GHD mention that the GHRH–arginine test can be misleading in those who received HP irradiation.^19^ In 2018, the Endocrine Society’s guidelines on hypothalamic-pituitary and growth disorders in survivors of childhood cancer recommend against the use GHRH alone or in combination with arginine in survivors treated with radiotherapy involving the HP axis; however, this recommendation is based on low quality evidence.^18^ In contrast, tests such as the insulin tolerance test (ITT) and glucagon stimulation test (GST), which are believed to stimulate the entire hypothalamic-pituitary axis, are recommended by expert consensus to be used in this population.^18^

In children, most of the evidence for GH stimulation tests comes from patients presenting with short stature. Validation studies have focused on distinguishing true GHD from normal variants, such as familial short stature or constitutional delay of growth and puberty.^32–35^ Among children diagnosed with GHD, the most frequently identified aetiology is idiopathic.^36^ Again, this pathophysiology differs from that seen in individuals treated with radiotherapy.

Despite their widespread clinical use in patients exposed to radiotherapy, the diagnostic accuracy of GH stimulation tests in this group is not well documented. Much of the current evidence seems to be extrapolated from populations with fundamentally different disease mechanisms. To address this gap, we aim to conduct a systematic review and meta-analysis where feasible, to assess the diagnostic accuracy of GH stimulation tests for GHD specifically in children, teenagers and young adults (TYA) and adult tumour survivors following radiotherapy involving the HP axis. To our knowledge, this will be the first review to comprehensively assess these tests in this ever-growing population. We anticipate that the findings will help clinicians caring for cancer survivors select the most appropriate and validated test to diagnose GHD, thereby reducing the risk of misdiagnosis.

## Methods and analysis

### Eligibility criteria

Studies will be selected according to the following criteria.

#### Study characteristics

Eligible study designs include randomised controlled studies, controlled non-randomised studies, prospective or retrospective comparative observational studies, cross-sectional studies and case series. Reviews, meta-analyses, case reports, conference abstracts, editorials, commentaries and letters will be excluded.

Studies must enrol children (<18 years old), TYA (18–24 years old) or adults (>24 years old) who were exposed to radiotherapy involving the HP axis for any indication, and who subsequently underwent GH stimulation testing for suspected GHD in any clinical setting. We will exclude studies only reporting that some participants received radiotherapy, without providing separate diagnostic test results for irradiated patients or distinguishing outcomes between irradiated and non-irradiated populations.

We will include studies evaluating stimulation tests commonly used in clinical practice, recommended in guidelines or supported by emerging evidence. In children, these include the ITT, GST, arginine stimulation test, clonidine stimulation test and macimorelin stimulation test.^37^ In TYA and adults, eligible tests include the ITT, GST, GHRH-arginine stimulation test and macimorelin stimulation test.^16 19 38 39^ Studies assessing other stimulation tests will be excluded unless they also evaluate at least one of the eligible tests. Studies using different diagnostic cut-offs for the same test will be included and data about cut-offs will be described.

The reference standards can either be clinical or biochemical. A clinical reference standard will be defined as the presence of ≥2 additional pituitary hormone deficiencies (other than GHD), as previous studies suggest that approximately 90% of such patients have severe GHD.^40 41^ A biochemical reference standard is another GH stimulation test and can be one of the following: ITT, GST, arginine stimulation test or clonidine stimulation test in children; ITT or GST in adults. We acknowledge that some of these tests may function both as index tests and as reference standards across studies. This reflects the absence of a perfect gold standard for diagnosing GHD in our population of interest.

To be included, studies must provide sufficient information, either directly or indirectly, to derive a 2×2 contingency table for our target population.

#### Report characteristics

Included studies must present original data and be published in peer-reviewed journals. There will be no restrictions based on setting, geographical location or year of publication. Only full-text articles published in English, French and Spanish will be included.

### Information sources and search strategy

A Population, Intervention, Comparison, and Outcome framework was used to break down the research question into three core concepts. These concepts included growth hormone deficiency, diagnostic tests and diagnostic accuracy. We compiled search terms for each concept and combined them using Boolean operators (AND/OR). Additional MeSH and EMTree terms were included to maximise the efficiency of the strategy. To exclude bench science and animal studies, a list of exclusion terms was compiled and combined using NOT operators. Additionally, results have been limited to articles published in English, French and Spanish. No other restrictions will be applied. No start date limit will be applied, and the search will include publications up to 1 July 2026. To ensure comprehensive coverage of the literature, we will examine the reference lists of included studies identified through the search. The authors’ personal files will also be reviewed to identify any further relevant studies.

The search strategy has been developed in collaboration with a health sciences librarian with expertise in systematic review searching. The search strategy has then been peer-reviewed by two experienced and independent librarians. We plan to conduct the search across four healthcare databases: MEDLINE (OVID interface), Embase (OVID interface), The Cochrane Central Register of Controlled Trials (Wiley interface) and PubMed (National Library of Medicine). The MEDLINE search strategy is presented in table 2. This search strategy will be adapted to match the format and indexing terms of the other databases.

**Table 2:** MEDLINE search strategy

#▲	Searches
1	((“Growth Hormone” or “gh”) adj2 (Deficienc* or insufficienc* or lack* or deficit or short* or stimulation)).ab,kw,ti.
2	(“Insulin Tolerance” or ITT or Macimorelin or Ghrelin or “Glucagon stimulation” or Arginine or Clonidine or “Growth Hormone Releasing Hormone” or GHRH).ab,kw,ti.
3	Ghrelin/ or Glucagon/ or Arginine/ or Clonidine/ or Growth Hormone-Releasing Hormone/
4	2 or 3
5	((area adj4 curve) or Sensitiv* or specific* or “recall rate” or “ROC Curve” or “Predictive Value” or “Signal to Noise Ratio” or perform* or Diagnos*).ab,kw,ti.
6	Sensitivity and Specificity/
7	5 or 6
8	1 and 4 and 7
9	(nonhuman or non-human or animal* or mice or mouse or “basic science” or “bench science” or Arthropod* or Cestode* or Nematode* or Trematode* or Bacterium or Filaria or Microorganism* or Protozoon* or Virus or Algae or lichen* or mosses or fern* or Fungus or Fungi or Higher plant or Plant or Animal* or Amphibia* or Ape* or Bird* or Cat or Cattle or Chicken* or Dog* or Duck* or Geese or Fish* or Frog* or Toad* or Goat* or Guinea pig* or Hamster* or Gerbil* or Horse* or Monkey* or Mouse or Mice or Pigeon* or Dove* or Rabbit* or Hare* or Rat or Reptile* or Sheep or Swine* or Animal Studies or pig or pigs).ab,ti.
10	8 not 9
11	limit 10 to (English or French or Spanish)

### Study records

Results from all databases will be merged and initially deduplicated using the RefWorks reference manager. Records will then be imported into Rayyan.ai, a web-based platform that facilitates collaboration during the study selection process, where any remaining duplicates will be identified and removed. Title and abstracts will be screened independently by two reviewers using Rayyan.ai. Studies deemed potentially eligible at this stage will be selected for full-text review, while those clearly not meeting inclusion criteria will be excluded. Full-text articles of potentially eligible studies will be assessed independently by both reviewers. Reasons for exclusion will be documented. Disagreements will be solved through discussion with a third member of the research team. All screening decisions and records will be managed using Rayyan.ai. Reference lists of included studies will be examined to identify any additional eligible studies. The number of records identified, screened, included and excluded at each stage will be summarised using a Preferred Reporting Items for Systematic Reviews and Meta-Analyses (PRISMA) flow diagram.

### Data collection

Data will be collected using a standardised form with detailed instructions. Prior to full data extraction, the process will be piloted to maximise consistency across reviewers. Two reviewers will independently extract data from each eligible study. Any disagreements will be resolved through discussion, with a third member of the research team consulted if needed.

We will extract the following data:

- General information: Authorship, title, year of publication, journal, country of conduct, funding sources and conflicts of interest.
- Methodology: Study design, setting.
- Patients baseline characteristics: Number, age, age group, sex, Tanner stage in children and TYA, ethnicity, socio-economic status, body mass index (BMI), abdominal circumference, presence of sign and symptoms of GHD and reported quality of life scores.
- Radiotherapy details: Age at exposure; year of exposure; time since exposure; type and location of tumour; radiotherapy field; photon (three field, intensity modulated radiotherapy, volumetric modulated arc or whole brain) radiotherapy versus proton radiotherapy, radiation dose (Gy) to the hypothalamus, pituitary and HP axis; total dose (Gy); and fractionation schedule.
- Control group (if applicable): Method of control group selection, matching or adjustment factors and the same baseline characteristics as described for the patients. If control group was also exposed to radiotherapy, the same radiotherapy details as above will be extracted.
- IGF-1 data: Year of testing, type of IGF-1 assay, reference ranges, reporting format (Z score vs percentage of the upper limit of normal), reported value and units.
- Index test(s): Year of testing, type of GH stimulation test, route of administration, dose, sampling intervals, total test duration, diagnostic cut-off(s), rationale for selection of the cut-off threshold, assay used for GH measurement, use of sex hormones priming (for children and TYA) and regimen type, absolute GH peak and nadir results, units used for GH reporting and reported side effects (frequency and type).
- Reference standard: Type of reference standard used and the rationale for its selection. If the reference standard is also a stimulation test, the same technical characteristics as described for the index test(s) will be extracted.
- Diagnostic accuracy outcomes: Number of true positives, false positives, true negatives, false negatives, sensitivity, specificity and area under the receiver operating characteristic curve.
- Reporting quality: Whether the manuscript adheres to the STARD checklist.

When important information is missing, study authors will be contacted by email, with up to three attempts. If essential data cannot be obtained, the study will be excluded.

### Outcomes

The primary outcomes will be the sensitivity and specificity of GH stimulation tests for GHD in individuals exposed to radiotherapy involving the HP axis.

Secondary outcomes will include:

1. Investigation of heterogeneity: Exploration of whether diagnostic accuracy and diagnostic cut-offs differed according to age category, BMI, ethnicity, dose of radiotherapy and timing of GH stimulation testing after radiotherapy involving the HP axis. Radiotherapy is thought to initially damage the hypothalamus, with secondary pituitary atrophy developing over time.^42 43^ The timing of testing is therefore critical, as some GH stimulation tests primarily assess pituitary function. For these tests, performing them too soon after radiotherapy may yield false-negative results, whereas later testing, once pituitary atrophy has occurred, may provide more accurate diagnostic information.
2. Diagnostic cut-offs: The rationale and methods used by study authors to define diagnostic cut-off values for GH stimulation tests.
3. Safety outcomes: Adverse effects and safety profile associated with GH stimulation tests when used as index tests or reference standards.
4. Methodological quality: Risk of bias and concerns regarding applicability of included studies, assessed using the Quality Assessment of Diagnostic Accuracy Studies-2 (QUADAS-2) tool.
5. Reporting quality: Compliance with the STARD guidelines.^23^

### Critical assessment

Risk of bias will be independently assessed by two reviewers using QUADAS-2 tool.^44^ QUADAS-2 is recommended by the National Institute for Health and Care Excellence, Cochrane and the Grading of Recommendations Assessment, Development and Evaluation (GRADE) for assessing methodological quality and risk of bias in diagnostic accuracy studies.^45 46^ A study will be considered as having ‘low risk of bias’ or ‘low concern regarding applicability’ if all relevant domains are rated as ‘low’. If one or more domains are rated as ‘high’ or ‘unclear’, the study will be considered at ‘risk of bias’ or as ‘having concerns regarding applicability’. Disagreements will be resolved through discussion, with a third member of the research team consulted if needed. Results will be summarised for all included studies across the four QUADAS-2 domains.

The overall certainty of the evidence will be assessed by two reviewers independently, using the GRADE approach. All reviewers have completed formal GRADE training and are familiar with the GRADE guidance for rating the certainty of evidence in diagnostic test accuracy reviews.^46–48^ The certainty of a body of evidence will be classified as high, moderate, low or very low. Certainty will initially be rated according to study design: evidence based mainly on one-gate design will start at high certainty, and evidence based mainly on two-gate design (diagnostic case–control study) will start at low certainty. The rating may then be downgraded based on risk of bias, inconsistency, indirectness, imprecision and publication bias. We anticipate some degree of inconsistency, given the potential heterogeneity across studies in patient populations, index test protocols, diagnostic cut-offs and reference standards. Where more than five studies are available for a given meta-analysis, publication bias will be assessed using Deeks’ test.^47 49^ Where five or fewer studies are available, the potential for publication bias will be assessed qualitatively.

An equality and impact assessment will also be conducted. In addition, we will evaluate how protected characteristics (such as age, sex, ethnicity and socio-economic status) are reported in the included studies, in order to assess whether these factors are adequately represented and considered in the existing literature.

### Data synthesis

Findings from the included studies will be summarised with a narrative synthesis. Forest plots of sensitivity and specificity will be presented, along with summary receiver operating characteristic (SROC) curves when appropriate. If the included studies are comparable in terms of index test procedures, diagnostic cut-offs and reference standards, we will undertake meta-analyses separately for each reference standard:

- Presence of ≥2 additional pituitary hormone deficiencies (other than GHD).
- ITT.
- GST.
- Arginine stimulation test (only in children).
- Clonidine stimulation test (only in children).

Pooled estimates of sensitivity and specificity will be calculated using hierarchical models for diagnostic test accuracy, such as the bivariate model and the hierarchical SROC model.

Where sufficient studies are available, studies using different diagnostic cut-offs will be analysed separately to account for differences in test thresholds. If there are too few studies or substantial differences between studies to allow meaningful comparison of cut-offs, the findings will be presented narratively rather than pooled.

Subgroup analyses are planned to explore potential sources of heterogeneity and will be performed only if sufficient studies are available.

The following subgroups are prespecified:

1. Age category:
  - Children: <18 years old.
  - TYA: 18–24 years old.
  - Adults: >24 years old.
2. Body mass index:
  - Children.
    - BMI standard deviation score (SDS) ≤+1 standard deviation (SD.
    - BMI SDS >+1 SD and ≤+2 SD.
    - BMI SDS >+2 SD.
  - TYA and adults.
    - BMI <25 kg/m².
    - BMI 25 to <30 kg/m².
    - BMI ≥30 kg/m².
3. Ethnicity:
  - Predominantly Caucasian populations.
  - Other or mixed ethnic populations.
4. Time since radiotherapy:
  - <5 years.
  - 5 to <10 years.
  - ≥10 years.
5. Dose of radiotherapy involving the HP axis:
  - ≤18 Gy.
  - >18 and <30 Gy.
  - ≥30 Gy.

We will conduct the following sensitivity analyses to assess the robustness of our findings:

1. Quality assessment according to the QUADAS-2 tool.
2. Study design.

### Patient and public involvement

Two patient and public involvement and engagement contributors who are cancer survivors were involved in the planning of this systematic review. The first one is a TYA cancer survivor, and the second one is an adult survivor. They were consulted to ensure that the research question is relevant and meaningful from a patient perspective, and that the objective of the review is clear and understandable. Both contributors confirmed that they considered the research question important and that the proposed approach was understandable.

## Ethics and dissemination

Ethical approval is not required as this systematic review summarises published data only. We will share our findings through peer-reviewed publications, conference presentations and engagement with relevant clinical teams to improve diagnostic decision-making in our population of interest.

### Registration

In accordance with the guidelines, our systematic review protocol was registered with the International Prospective Register of Systematic Reviews (PROSPERO) on 30 June 2026 (registration number: CRD420261415689).
