# Supplementary material for: Diagnostic accuracy of growth hormone stimulation tests for growth hormone deficiency following radiotherapy involving the hypothalamic-pituitary axis: protocol for a systematic review and planned meta-analysis
Source: BMJ Open. 2026 Sep 21;16(9):e125247. doi: 10.1136/bmjopen-2026-125247 (PMC13599972; doi:10.1136/bmjopen-2026-125247)
Supplement: Supplementary file 1 [file bmjopen-2026-125247.reviewer_comments.pdf]

PEER REVIEW HISTORY

BMJ Open publishes all reviews undertaken for accepted manuscripts. Reviewers are asked to complete a checklist review form and are provided with free text boxes to elaborate on their assessment. These free text comments are reproduced below.

ARTICLE DETAILS

Title (Provisional)

Diagnostic Accuracy of Growth Hormone Stimulation Tests for Growth Hormone Deficiency Following Radiotherapy Involving the Hypothalamic-Pituitary Axis: Protocol for a Systematic Review and Planned Meta-analysis

Authors

Chen, Lillian Ruiheng; Perry, Melissa Betty; Salomon Estebanez, Maria; Livesey, Daniel; Martin, Glen Philip; Higham, Claire E

VERSION 1 - REVIEW

**Reviewer**	**1**
**Name**	**Shen, Yanqin**
**Affiliation**	**Hangzhou Red Cross Hospital**
**Date**	**01-Aug-2026**
**COI**	**None**

The protocol demonstrates a good understanding of the complexities of DTA reviews. The use of QUADAS-2 for risk of bias assessment, the GRADE approach for certainty of evidence, and the plan for hierarchical bivariate and SROC models are all appropriate. The prespecified subgroup analyses are clinically relevant. However, this protocol still has some aspects that need improvement.

The protocol allows two types of reference standards: (a) clinical – presence of ≥2 additional pituitary hormone deficiencies (excluding GHD), and (b) biochemical – another GH stimulation test (e.g., ITT, GST). Some of these biochemical tests are also used as index tests. This is a classic “reference standard dilemma” in DTA reviews, but the protocol does not adequately address how this will be handled in the meta-analysis.

The protocol states that studies using different cut-offs for the same test will be included and “data about cut-offs will be described.” It does not specify how multiple cut-offs from different studies will be handled in the meta-analysis.

The search is limited to English, French, and Spanish. While this reduces language bias to some extent, it still excludes studies published in other languages (e.g., German, Chinese,

Japanese). This is a pragmatic choice but should be acknowledged as a potential source of bias. How translation will be undertaken.

The protocol mentions assessment of meta-bias in the PRISMA-P checklist but does not describe how publication bias will be assessed in the methods section.

VERSION 1 - AUTHOR RESPONSE

1. **The protocol allows two types of reference standards: (a) clinical – presence of ≥2 additional pituitary hormone deficiencies (excluding GHD), and (b) biochemical – another GH stimulation test (e.g., ITT, GST). Some of these biochemical tests are also used as index tests. This is a classic "reference standard dilemma" in DTA reviews, but the protocol does not adequately address how this will be handled in the meta-analysis.**

   We thank the reviewer for highlighting this important point. We have clarified in the "Methods" section that meta-analyses will be stratified by reference standard, rather than pooling clinical and biochemical reference standards together (see page 18). The prespecified reference standards are:

   - Presence of ≥ 2 additional pituitary hormone deficiencies (other than GHD)
   - ITT
   - GST
   - Arginine stimulation test (only in children)
   - Clonidine stimulation test (only in children)

   These were previously listed as subgroup analyses. Following the reviewer's comment, we have changed this to a prespecified stratification of the primary meta-analysis. We recognise that the use of different reference standards, along with other sources of heterogeneity across studies, may limit the feasibility of meta-analysis. Where quantitative pooling is not appropriate, the findings will instead be presented as a narrative synthesis. We consider this an important and valuable approach in its own right, as it will allow a comprehensive assessment of the available evidence and provide useful information to clinicians even when a pooled estimate cannot be obtained.

2. **The protocol states that studies using different cut-offs for the same test will be included and "data about cut-offs will be described." It does not specify how multiple cut-offs from different studies will be handled in the meta-analysis.**
   We thank the reviewer for this comment. We have added to the "Methods" section that, where sufficient studies are available, studies using different diagnostic cut-offs will be analysed separately to account for differences in test thresholds (see page 18). If there are too few studies or substantial differences between studies to allow meaningful comparison of cut-offs, the findings will be presented narratively rather than pooled.

3. **The search is limited to English, French, and Spanish. While this reduces language bias to some extent, it still excludes studies published in other languages (e.g., German, Chinese, Japanese). This is a pragmatic choice but should be acknowledged as a potential source of bias. How translation will be undertaken.**

   We have added the inclusion of only three languages as a potential source of bias in the "Strength and Limitation of this study" (see page 4). These three languages were selected because members of the review team are fluent in English, French and Spanish and can therefore assess studies in these languages without relying on translation. In addition, a previous study has found that "Restricting systematic reviews to English-language publications appears to have little impact on the effect estimates and conclusions of systematic reviews."[1]

4. **The protocol mentions assessment of meta-bias in the PRISMA-P checklist but does not describe how publication bias will be assessed in the methods section.**

   We thank the reviewer for highlighting this. We agree that the protocol did not previously provide sufficient detail on how publication bias would be assessed. We have now clarified this in the "Methods" section. Where more than five studies are available for a given meta-analysis, publication bias will

be assessed using Deeks' test[2 3] (see page 17). Where five or fewer studies are available, the potential for publication bias will be assessed qualitatively.

Sources

1. Dobrescu AI, Nussbaumer-Streit B, Klerings I, et al. Restricting evidence syntheses of interventions to English-language publications is a viable methodological shortcut for most medical topics: a systematic review. *J Clin Epidemiol* 2021;137:209-17. doi: 10.1016/j.jclinepi.2021.04.012 [published Online First: 20210430]
2. van Enst WA, Ochodo E, Scholten RJ, et al. Investigation of publication bias in meta-analyses of diagnostic test accuracy: a meta-epidemiological study. *BMC Med Res Methodol* 2014;14:70. doi: 10.1186/1471-2288-14-70 [published Online First: 20140523]
3. Schunemann HJ, Mustafa RA, Brozek J, et al. GRADE guidelines: 21 part 2. Test accuracy: inconsistency, imprecision, publication bias, and other domains for rating the certainty of evidence and presenting it in evidence profiles and summary of findings tables. *J Clin Epidemiol* 2020;122:142-52. doi: 10.1016/j.jclinepi.2019.12.021 [published Online First: 20200210]
